# Comparative Hepatoprotective Effects of Esculetin and Its Derivatives Against Oxidative Stress

**DOI:** 10.3390/antiox14070787

**Published:** 2025-06-26

**Authors:** Yoonjeong Kim, Jihyun Kwon, Jae-Hwan Kwak, In-hwan Baek, Younghwa Kim

**Affiliations:** 1 Department of Food Science and Biotechnology, Kyungsung University, Busan 48434, Republic of Korea; ang1569@naver.com (Y.K.); kgh0774@naver.com (J.K.); 2 College of Pharmacy, Chungbuk National University, Cheongju 28160, Republic of Korea; jhkwak@chungbuk.ac.kr; 3 College of Pharmacy, Kyungsung University, Busan 48434, Republic of Korea; baek@ks.ac.kr; 4 Functional Food & Drug Convergence Research Center, Industry-Academic Cooperation Foundation, Kyungsung University, Busan 48434, Republic of Korea

**Keywords:** esculetin, derivatives, antioxidant activity, ROS, HepG2 cells

## Abstract

In this study, we evaluated the antioxidant activities of esculetin and four synthesized derivatives (E1, 2-oxo-2H-1-benzopyran-6,7-diyl diacetate; E2, 7-hydroxy-2-oxo-2H-1-benzopyran-6-yl acetate; E3, 7-(methoxymethoxy)-2-oxo-2H-1-benzopyran-6-yl acetate; E4, 7-hydroxy-2-oxo-2H-1-benzopyran-6-yl 2,4-dinitrobenzene-1-sulfonate) against oxidative stress in hepatocytes. In HepG2 cells, treatment with 1 mM *tert*-butyl hydroperoxide (TBHP) reduced cell viability to 40%, while co-treatment with esculetin restored cell viability. Among the esculetin derivatives, E2 exhibited the most significant cytoprotective effect, while E4 showed the lowest. Furthermore, E2 at 25 µM concentration showed the similar effects to esculetin in reducing ROS generation and preventing glutathione depletion. The treatment of E2 also enhanced the expression of HO-1 and GCLC proteins against oxidative stress. On the other hand, TBHP-induced oxidative stress decreased antioxidant activities including glutathione reductase, glutathione peroxidase, and catalase; however, E2 significantly increased these antioxidant activities. These findings suggest that the esculetin derivative, particularly E2, possesses potential as an antioxidant aimed at enhancing physiological functions.

## 1. Introduction

Reactive oxygen species (ROS) are reactive chemicals that act as powerful regulators of various cellular activities, including gene expression and immune responses [1]. While ROS is essential for cellular functions, excessive production can lead to oxidative stress, which is responsible for damage to biomolecules such as DNA and proteins. This damage is directly linked to the initiation and progression of several diseases, including cancer, diabetes, neurodegenerative disorders, and aging [2,3]. Antioxidants can help inhibit oxidative damage and potentially reduce the risk of these related diseases [4]. Regular dietary intake of antioxidant-enriched foods has been widely linked to a decreased likelihood of developing chronic diseases, such as cancer and heart disease [5]. The liver, due to its crucial functions in metabolism and detoxification, is particularly susceptible to oxidative injury from both exogenous and endogenous factors [6]. Overproduction of ROS within the hepatocytes can trigger lipid peroxidation, mitochondrial damage, and apoptosis, and lead to hepatic disease, such as hepatitis, fibrosis, and hepatocellular carcinoma [7].

Esculetin (6,7-dihydroxycoumarin), a naturally occurring coumarin derivative present in foods such as lemons and citrus peels, exhibits significant pharmacological and biochemical activities [8]. Interestingly, esculetin has shown an ability to protect cells by neutralizing ROS across different cell types and inducing apoptosis in cancer cells by impacting their growth cycle [9]. Given these promising properties, researchers have tried to improve esculetin’s efficacy through structural modification. Some of the modified forms, with substitutions at C4 and C8 positions, have been shown to enhance antiproliferative activity and metabolic stability [10]. Glycosylation of derivatives, such as esculin, also exhibits improved antioxidant and hepatoprotective activity, possibly due to increased solubility and availability [11,12]. Thus, the design of esculetin derivatives has become a viable strategy to improve the therapeutic potential of the compound. To enhance the pharmacological potential of bioactive compounds, their chemical structures have been extensively altered [13]. The target derivatives were found to possess better pharmacokinetic profiles and, in most instances, better therapeutic activity than esculetin itself, particularly in oxidative stress models [14]. However, despite the known antioxidant potential of esculetin, the cytoprotective effects and mechanisms of its structural derivatives have not been fully characterized. This study aims to evaluate and compare the antioxidant and cytoprotective effects of selected esculetin derivatives in a TBHP-induced oxidative stress model using HepG2 cells in order to identify compounds with improved protective activity.

## 2. Materials and Methods

### 2.1. Chemicals

**Figure 1:**
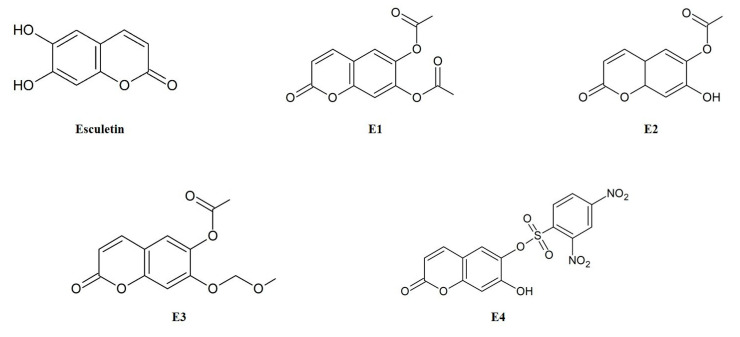
Structures of esculetin and its derivatives (E1–E4).

Esculetin derivatives (E1, 2-oxo-2H-1-benzopyran-6,7-diyl diacetate; E2, 7-hydroxy-2-oxo-2H-1-benzopyran-6-yl acetate; E3, 7-(methoxymethoxy)-2-oxo-2H-1-benzopyran-6-yl acetate; E4, 7-hydroxy-2-oxo-2H-1-benzopyran-6-yl 2,4-dinitrobenzene-1-sulfonate) were synthesized in Dr. Kwak’s laboratory and kindly provided for our research (Figure 1). Esculetin, trichloroacetic acid, 3-(4,5-dimethylthiazol-2-yl)-2,5-diphenyl-tetrazolium bromide (MTT), 2,7-dichlorofluorescin diacetate (DCFH-DA), and *tert*-butyl hydroperoxide (TBHP) were purchased from Sigma Chemical Co. (St. Louis, MO, USA). Dulbecco’s modified Eagle’s medium (DMEM), phosphate-buffered saline (PBS), fetal bovine serum (FBS), bovine serum (BS), trypsin-EDTA, and penicillin–streptomycin were obtained from Gibco (Lafayette, CO, USA). The reagents and solvents used in the assay were of the high-performance liquid chromatography grade.

### 2.2. Cell Culture

HepG2 cells were obtained from the American Type Culture Collection (ATCC, Manassas, VA, USA) and were cultured in high-glucose DMEM supplemented with 10% fetal bovine serum (FBS) and 1% penicillin–streptomycin, maintained at 37 °C under 5% CO_2_ conditions.

### 2.3. Cytotoxicity and Cytoprotective Effects

For cytotoxicity evaluation, HepG2 cells were seeded into 96-well plates at a density of 3×10^4^ cells/well and allowed to adhere for 24 h. MTT solution was then added, followed by incubation for 3 h at 37 °C. After discarding the medium, formazan crystals were dissolved using dimethyl sulfoxide, and absorbance was measured at 550 nm using a microplate reader (Thermo Scientific Ltd., Lafayette, CO, USA).

For the cytoprotective assays, HepG2 cells were plated at 1.5 × 10^4^ cells/well and incubated for 24 h. The culture medium was then replaced with FBS-free DMEM containing 1 mM TBHP and various concentrations of the samples (12.5, 25, 50, and 100 µM). After 6 h of treatment, cell viability was assessed using the MTT assay, following the same procedure as described above.

### 2.4. Measurement of ROS Generation

Intracellular ROS levels were evaluated using the DCFH-DA fluorescence method. HepG2 cells were seeded in 96-well black plates at a density of 1 × 10^5^ cells/well and pre-treated with either esculetin, which was used as the positive control, or the E2 derivative at concentrations of 12.5 or 25 µM. After the pretreatment, cells were exposed to 1 mM TBHP for 1 h, followed by incubation with 25 μM DCFH-DA in FBS-free DMEM for 1 h at 37 °C. The cells were washed with PBS before the addition of Hank’s balanced salt solution. After washing with PBS, fluorescene intensity was measured using a microplate reader with excitation at 485 nm and emission at 530 nm over a 2 h period.

### 2.5. Determination of Glutathione Levels and Antioxidant Enzyme Activities

HepG2 cells were treated with esculetin (positive control) or the E2 derivative, followed by exposure to 1 mM TBHP for 6 h. After treatment, the levels of glutathione (GSH) and the activities of antioxidant enzymes were measured. The activities of GSH, catalase (CAT), glutathione reductase (GR), and glutathione peroxidase (GPx) were quantified using previously reported protocols [15].

### 2.6. Western Blotting Analysis

The expression of antioxidant-related proteins, including glutamate–cysteine ligase catalytic subunit (GCLC) and heme oxygenase-1 (HO-1), was assessed by Western blot analysis as described previously [15]. Esculetin was used as a positive control in all experiments. Protein bands were visualized using ECL™ detection reagents (GE Healthcare, Buckinghamshire, UK), and protein levels were quantified using ImageJ software version 1.54k (NIH, Bethesda, MD, USA).

### 2.7. Statistical Analysis

Statistical analysis was performed using SAS software, version 9.4 (SAS Institute Inc., Cary, NC, USA). One-way analysis of variance (ANOVA) followed by Duncan’s multiple range test was used to determine significant differences between groups. A *p*-value of less than 0.05 was considered statistically significant.

## 3. Results and Discussion

### 3.1. Effects of Esculetin and Its Derivatives on Cytotoxicity and Cytoprotection Against Oxidative Stress

**Figure 2:**
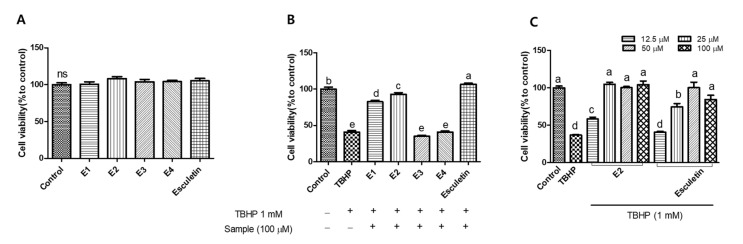
Cell viability (**A**) and cytoprotective effect (**B**) of esculetin and its derivatives (100 µM). Cytoprotective effect of esculetin and E2 on tert-butyl hydroperoxide (TBHP)-induced oxidative stress (**C**). Esculetin was used as a positive control in all experiments. Different letters above the bars indicate significant differences based on the Duncan’s multiple range test (*p* < 0.05). ns, not significant.

To investigate how specific structural modifications influence antioxidant and cytoprotective activity, a set of esculetin derivatives was synthesized with substitutions at the 6- and/or 7-hydroxyl positions. These modifications were introduced to probe the role of the catechol moiety and to assess the effects of varying electronic, steric, or hydrophilic properties. Previous studies have highlighted that such substitutions can significantly alter the biological activity of coumarin-based antioxidants [16,17]. In addition, several studies have reported that acetylation of phenolic compounds and coumarins improves their lipophilicity, membrane permeability, and overall biological activity by enhancing cellular uptake and bioavailability [18,19]. Accordingly, this study aimed to evaluate how structural modifications of esculetin such as acetylation and substitutions at hydroxyl positions affect cellular protection against oxidative stress. Figure 2A shows the cytotoxicity of esculetin and its derivatives. HepG2 cells were treated with the samples at a concentration of 100 μM and 1 mM TBHP for 6 h. None of the samples showed cytotoxic effects in comparison to the control group. Next, we evaluated the cytoprotective effects of esculetin and its derivatives against TBHP-induced oxidative damage (Figure 2B). Treatment with TBHP alone decreased cell viability by approximately 40%. However, esculetin, E1 and E2 significantly increased cell viability. Among the tested samples, esculetin showed the highest cell viability, while E3 and E4 did not possess significant cytoprotective effects compared to the other derivatives. Among esculetin derivatives, E2 was the most effective in protecting against oxidative stress in HepG2 cells. To further explore the cytoprotective effects, we used esculetin and E2 to investigate the antioxidant capacity. As shown in Figure 2C, treatment with esculetin and E2 increased the cell viability in HepG2 cells against TBHP. In particular, the 100 µM and 50 µM of both esculetin and E2 fully protected the cells from oxidative stress, showing no significant difference compared to the control group. However, 25 µM of E2 provided 100% protection against oxidative stress, while esculetin at the same concentration exhibited an approximately 70% protective effect, indicating that E2 exerted 1.4-fold higher cytoprotective activity than esculetin. Based on these findings, subsequent experiments were conducted using 25 μM and 12.5 μM concentrations.

Esculetin contains a catechol group with hydroxyl substituents at the 6- and 7-positions of its benzene ring. These hydroxyl groups are thought to contribute to esculetin’s antioxidant properties [20]. In fact, esculetin has been widely used as a reference antioxidant in cellular oxidative stress models, and was therefore used as the positive control in this study. Esculetin has been associated with cellular protection against oxidative stress in various in vitro models [21,22,23]. In studies with TBHP-treated HEK293 cells, esculetin has been shown to effectively reduce oxidative stress and improve cell viability, likely due to decreased levels of ROS [24]. The same protective effects have also been noticed in human leukemia NB4 cells, where esculetin reduced TBHP-induced cytotoxicity and apoptosis [25]. The biological activity of coumarin derivatives depends on the number of hydroxyl groups with the substituent’s position and type [26,27]. Some of the esculetin derivatives, such as methylated coumarin and 5,7-hydroxycoumarin, were observed to be more active antioxidants than esculetin [28]. The catechol moiety of esculetin is responsible for electron donation and ensuring the formation of a stable ortho-quinone structure [29,30]. The E2 derivative discussed in this study has an acetate ester substitution at the 6-hydroxyl group and a hydroxyl group at position 7. E2 exhibited stronger cytoprotective effects than esculetin under TBHP-induced oxidative stress, especially at low concentrations (25 μM). These findings support earlier studies regarding structural modifications affecting antioxidant efficacy. Several in vitro and model membrane studies support the notion that esterification of phenolic compounds can enhance their antioxidant activity and membrane interaction. Notably, previous studies have shown that resveratrol butyrate esters enhance reactive oxygen species scavenging and improve cell viability in HepG2 cells compared to unmodified resveratrol [31]. Similarly, Tain et al. (2020) reported that esterified resveratrol derivatives effectively reduced lipid accumulation in HepG2 cells [32]. These results suggest that the esterification of esculetin, as observed in the E2 compound, may contribute to its improved cellular activity under oxidative stress conditions. In addition, it has been demonstrated that protocatechuic acid ethyl ester exhibits enhanced incorporation into phospholipid bilayers in multilamellar vesicles and monolayers, supporting the notion that esterification can strengthen membrane interactions [33]. In addition, esterification of quercetin has been found to facilitate its transport across Caco-2 cell monolayers, indicating that this modification may aid intracellular delivery in certain phenolic compounds by increasing lipophilicity or altering membrane affinity [34]. Taken together, these findings suggest that the acetate ester group in E2 may have promoted membrane partitioning and cellular uptake, thereby contributing to its enhanced cytoprotective effects in HepG2 cells. For instance, acetylated flavonoids such as tetraacetylated quercetin have shown improved cellular uptake and antioxidant capacity in hepatic cell models [35]. Moreover, *O*-acetylated flavonoids have shown improved cellular uptake and antioxidant activity in cancer cells, highlighting the functional benefits of acetylation in phenolic compounds [36]. These findings support that acetylation at specific positions, including the 6-position in E2, can play a beneficial role in modulating cellular activity. In addition, dihydroxylated coumarins such as daphnetin and fraxetin with vicinal diols have been characterized as having high antioxidant activities and performing free radical scavenging through various mechanisms [37]. Esculin, represented by the sugar-coupled form of esculetin with an added sugar moiety at the 6-position carbon, has been indicated to stimulate Nrf2 signaling with an increase in antioxidant protein expression levels, including GPx and GR [38]. Other studies have presented that coumarin derivatives with amide or heterocycle groups have improved bioavailability with increased antioxidant and anti-inflammatory effects [39,40]. These findings emphasize the beneficial influence of modifications in esculetin’s structural framework in its cytoprotective efficacy in oxidative stress conditions. These examples, including E2, emphasize the beneficial impact of targeted structural modifications in improving antioxidant performance in cellular systems. However, the specific mechanisms by which these structural modifications increase antioxidant activity have not yet been fully delineated. More studies on the structure–activity relationship are required to enable full appreciation of how these substituents impact bioactivity.

### 3.2. Effects of Esculetin and Its Derivatives on Intracellular ROS Accumulation and GSH Depletion

**Figure 3:**
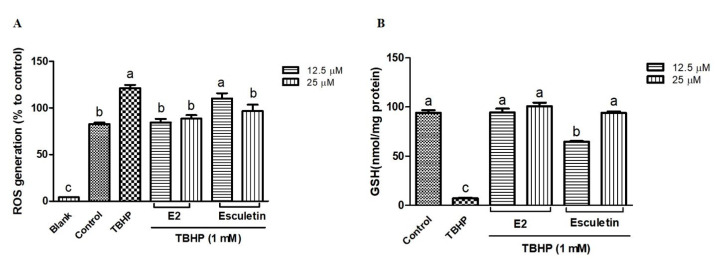
Effect on tert-butyl hydroperoxide (TBHP)-induced intracellular reactive oxygen species (ROS) formation (**A**) and GSH depletion (**B**) of esculetin and E2 derivatives. Esculetin was used as a positive control. Different letters above the bars indicate significant differences based on the Duncan’s multiple range test (*p* < 0.05).

In order to assess their protective effects, the influence of esculetin and its E2 derivative on TBHP-induced ROS production and GSH depletion was evaluated in HepG2 cells. As shown in Figure 3A, treatment with TBHP (1 mM) significantly increased ROS generation compared to the untreated control group (*p* < 0.05). Pretreatment with esculetin and E2 derivatives at both 12.5 μM and 25 μM concentrations led to a reduction in ROS levels, indicating their antioxidant potential. Notably, E2 exhibited a significantly stronger ROS scavenging effect than esculetin at 12.5 µM. At this concentration, esculetin reduced ROS levels by approximately 8.9%, whereas E2 achieved a reduction of about 30.6% relative to TBHP-treated cells (Figure 3A). These results indicate that E2 possesses superior antioxidant potential under the given experimental conditions, with statistically significant differences between the two compounds (*p* < 0.05). In parallel, GSH levels were assessed as a marker of intracellular redox balance (Figure 3B). TBHP markedly depleted intracellular GSH content, consistent with previous reports that TBHP induces oxidative stress by promoting lipid peroxidation and depleting thiol-based antioxidants such as GSH [41]. However, cells pretreated with esculetin or the E2 derivative exhibited significant restoration of GSH levels, suggesting a cytoprotective mechanism that may involve the preservation or upregulation of endogenous antioxidant defenses. At 12.5 µM, E2 restored GSH levels to nearly 94% of control values, whereas esculetin restored them to approximately 64%, suggesting that E2 may have a stronger influence on maintaining intracellular redox balance.

This observation is in agreement with a previous report of the antioxidative activities of esculetin, and it has been demonstrated to scavenge directly for the free radicals and also modulate the cellular antioxidant enzyme systems [42]. Esculetin’s phenolic structure is believed to contribute to its radical-scavenging activity, while structural modifications in E2 derivatives may affect their reactivity and cellular uptake [43]. Catechol group of esculetin is considered a key pharmacophore for efficient radical scavenging activities [38]. In addition, compounds with potent anti-inflammatory activity against TNF-α and IL-6 were obtained from the cycloaddition reaction of 4-chloro-2-oxo-2H-chromene-3-carbaldehydes with activated alkynes [44]. These results suggest that E2 (6-hydroxy-2-oxo-2H-chromene-3-carbaldehyde) have the potential to alter intracellular antioxidant activity. More recent studies related to the structure–activity relationship of esculetin and its analogs have also supported these observations, thus emphasizing the importance of the molecular structure in controlling antioxidant action. The high radical scavenging ability of esculetin is widely attributed to its phenolic structure, which both lowers ROS levels and increases GSH under oxidative stress [45]. The previous studies have shown that the 6,7-dihydroxy group, along with the coumarin derivatives, retains the highest ability to suppress ROS, and partial substitutions in the structure further increase intracellular antioxidant protection mechanisms [46]. Although E2 showed relatively lower ability in ROS scavenging compared to the widely used esculetin, it showed comparable or even better protection in the maintenance of intracellular GSH levels. This finding implies that structural modifications may have enabled specific antioxidant pathways from its parent molecule. Modifying functional groups on coumarin backbones has been demonstrated to impact physicochemical characteristics and enhance biological functionality in various biological matrices. For instance, Yang et al. (2022) proved that coupling coumarin moieties with carbohydrate backbones augmented antioxidant activity, suggesting that structural changes in coumarin may modulate redox potential [47]. Recent studies have also emphasized that substitutions on coumarin derivatives can significantly alter their functional groups, which may play a role in regulating antioxidant mechanisms at the cellular level [48]. However, further studies are needed to determine the effects of these changes in the substitutions on their structure–activity relationships.

### 3.3. Effects of Esculetin and Its Derivatives on the Antioxidant Enzyme Activities

**Figure 4:**
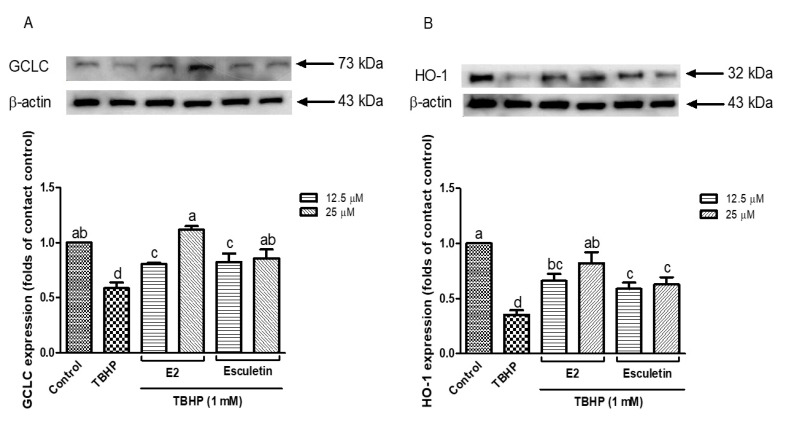
Expression of GCLC (**A**) and HO-1 (**B**) in esculetin and its derivatives treated cells. Esculetin was used as a positive control in all measurements. Different letters above the bars indicate significant differences based on the Duncan’s multiple range test (*p* < 0.05).

To further explore the cytoprotective mechanisms of esculetin and its E2 derivatives under oxidative stress, the protein expression levels of key antioxidants, including GCLC and HO-1 were examined. As seen in Figure 4A, GCLC expression was drastically inhibited after the administration of TBHP (1 mM) compared to the control group (*p* < 0.05), and it suggests the interruption of the cellular glutathione biosynthesis pathway. However, pretreatment with esculetin and E2 derivatives restored GCLC expression. In particular, E2 at 12.5 μM exhibited the most robust upregulation of GCLC, surpassing even the untreated control level, suggesting a concentration-dependent activation of the glutathione biosynthesis. Similarly, Figure 4B demonstrates the expression pattern of HO-1, a well-known stress-inducible enzyme that exerts antioxidant and anti-inflammatory effects. TBHP exposure markedly decreased HO-1 expression, consistent with previous studies that oxidative stress can dysregulate the Nrf2/ARE pathway [49].

Pretreatment with esculetin and E2 derivatives significantly restored HO-1 expression. Notably, esculetin at 25 μM appeared more effective in maintaining HO-1 levels compared to E2, though both compounds exhibited statistically similar protective effects. These results suggest that both esculetin and its E2 derivatives not only attenuate oxidative stress directly by scavenging ROS, but also exert indirect antioxidant effects by upregulating cytoprotective enzymes such as GCLC and HO-1. This supports previous findings that coumarin derivatives like esculetin activate the Nrf2 pathway, leading to enhanced expression of downstream antioxidant genes [50,51]. These results provide further evidence that esculetin and its E2 derivatives contribute to cellular redox homeostasis not only by mitigating oxidative damage but also by enhancing the expression of antioxidant enzymes critical to cellular defense.

To further elucidate the antioxidant potential of esculetin and its E2 derivatives, we assessed the activities of three key antioxidant enzymes—GR, GPx, and CAT—in TBHP-treated HepG2 cells (Table 1). The following results explain the influence of esculetin and its derivatives on the antioxidant enzyme activity of HepG2 cells. Treatment with TBHP resulted in a decline in GR activity. However, when treated with esculetin and its derivative E2, there was a significant increase in GR activity. The GSH levels were significantly depleted under oxidative stress but recovered following treatment with E2 and esculetin. Similarly, the TBHP-induced elevation in CAT activity was reduced by E2 treatment, returning to control levels. These results suggest that esculetin and its E2 derivative may modulate antioxidant enzyme systems under oxidative stress.

The observed increase in GR activity, along with the normalization of GPx and CAT levels, suggests that these compounds may enhance GSH regeneration and contribute to the restoration of redox balance [52]. This finding is consistent with previous studies reporting that phenolic compounds, such as esculetin, stimulate antioxidant defenses by scavenging free radicals and modulating the expression of antioxidant enzymes [53,54]. In addition, both esculetin and E2 significantly elevated the expression of GCLC and HO-1 under TBHP-induced oxidative stress conditions. This indicates that the coumarin derivatives could activate the defense mechanisms via Nrf2 pathway activation [55]. Esculetin enhances Nrf2 nuclear translocation and subsequently upregulates antioxidant gene expression, including HO-1 and GPx [56]. More recent research has also explored the role of modification to the coumarin backbone in antioxidant responses. Hydroxycoumarin derivatives with various substitutions have been found to exhibit different inhibition of the enzyme hydroxymethylglutaryl-CoA reductase and have varying antioxidant activities. This indicates that specific substitution patterns can affect redox-mediated mechanisms, such as the Nrf2 signaling pathway [57]. Previous studies have suggested that structural modifications to coumarin derivatives, particularly the addition of aldehyde or hydroxyl groups, can significantly boost the expression of HO-1 and GCLC, leading to enhanced cellular antioxidant defenses [50]. Other research also highlighted that incorporating electron-donating groups, such as methoxy or hydroxyl groups, and their specific positioning on the molecule can greatly improve radical scavenging activities [58]. These structure–activity relationships underscore the importance of functional groups, and electronic properties may impact a compound’s ability to regulate oxidative stress responses.

## 4. Conclusions

The current investigation evaluated the cytoprotective effects of esculetin, and its derivatives (E1-E4) against oxidative stress were assessed in HepG2 cells. The results showed that at a concentration of 100 µM, the samples did not show cytotoxicity. Treatment with TBHP, which induces oxidative stress, reduced cell survival to 40%; however, treatment with the samples led to an increase in cell survival. In particular, E2 showed strong cytoprotective effects at 25 µM. When evaluating the ability of esculetin and its derivatives to inhibit ROS formation in response to oxidative stress, E2 at 25 µM significantly reduced ROS formation and GSH depletion to levels similar to those of the control group. In addition, E2 increased both the HO-1 and GCLC protein expression and the activity of antioxidant enzymes against oxidative stress. These findings suggest that E2 has strong antioxidant and hepatoprotective potential, supporting its possible development as a functional food ingredient or for industrial applications. However, additional investigations are required to better elucidate the intracellular mechanisms influenced by these structural modifications.

## Figures and Tables

**Table 1:** Effect of esculetin and its derivatives on antioxidant enzyme activities in HepG2 cells.

Treatment		GR (nmol/min/mg of Protein)	GPx (nmol/min/mg of Protein)	CAT (μmol/min/mg of Protein)
Control		8.461 ± 1.200 ^c^	8.160 ± 1.133 ^d^	6.000 ± 0.470 ^c^
TBHP (1 mM)		4.335 ± 0.903 ^d^	27.222 ± 3.040 ^a^	6.622 ± 1.161 ^a^
TBHP (1 mM)	E2 (12.5 μM)	12.041 ± 1.454 ^a^	21.343 ± 2.464 ^b^	5.556 ± 0.427 ^b^
	E2 (25 μM)	10.238 ± 0.441 ^b^	14.145 ± 2.890 ^c^	5.156 ± 0.364 ^c^
	Esculetin (12.5 μM)	10.213 ± 1.032 ^b^	27.467 ± 2.479 ^a^	5.689 ± 0.524 ^a^
	Esculetin (25 μM)	10.006 ± 1.372 ^b^	13.528 ± 2.409 ^c^	5.289 ± 0.393 ^c^

Values are the means ± SD (n = 3). Different letters in the same column indicate significant difference (by ANOVA, *p* < 0.05). GR, glutathione reductase; GPx, glutathione peroxidase; CAT, catalase; TBHP, tert-butyl hydroperoxide. Esculetin was used as a positive control.

## Data Availability

The raw data supporting the conclusions of this article will be made available by the authors on request.

## Abbreviations

The following abbreviations are used in this manuscript:

BS	Bovine serum
CAT	Catalase
DMEM	Dulbecco’s modified Eagle’s medium
FBS	Fetal bovine serum
GPx	Glutathione peroxidase
GR	Glutathione reductase
GSH	Glutathione
MTT	3-(4,5-dimethyl-thiazol-2-yl)-2,5-diphenyl-tetrazolium bromide
PBS	Phosphate-buffered saline
ROS	Reactive oxygen species
TBHP	*tert*-Butyl hydroperoxide
